# Vicarious Menstruation

**Published:** 1882-05

**Authors:** 


### VICARIOUS MENSTRUATION.

An unusual case of vicarious menstruation is reported (*American Journal of Obstetrics,* April, 1882) by Dr. J. T. Gordon. The subject, a woman 41 years of age and weighing 254 pounds, bleeds "from the inner side of the thumb near the junction of the phalanges." The bleeding recurs monthly, lasts from three to five days, is pretty profuse, and is entirely painless. The bleeding has continued at the monthly periods for seven years with the exception of a period of about a year and a-half during which she had borne and nursed a child.

During the intervals between the bleedings, the spot is recognized by a slight blueness of the skin over an area not larger than a split pea.

